# The circulating 70 kDa heat shock protein (HSPA1A) level is a potential biomarker for breast carcinoma and its progression

**DOI:** 10.1038/s41598-022-17414-6

**Published:** 2022-07-29

**Authors:** Gabriela Boufelli de Freitas, Laura Penteado, Mila Meneguelli Miranda, José Roberto Filassi, Edmund Chada Baracat, Iara Moreno Linhares

**Affiliations:** Disciplina de Ginecologia, Departamento de Obstetrícia E Ginecologia, Hospital das Clínicas, Faculdade de Medicina, Universidade de São Paulo, São Paulo, Brazil

**Keywords:** Cancer, Biomarkers, Diseases, Medical research, Oncology

## Abstract

The early diagnosis of breast cancer can improve treatment and prognosis. We sought to evaluate whether the serum concentration of the 70 kDa heat shock protein (HSPA1A) was elevated in Brazilian women with breast cancer, and if levels correlated with tumor characteristics. This was a cross-sectional, analytical, case–control exploratory study performed at The University of São Paulo School of Medicine. From September 2017 to December 2018, 68 women with breast cancer and 59 controls were recruited. The HSPA1A concentration in serum samples was determined by ELISA by individuals blinded to the clinical data. The mean ages in the study and control groups were 54.9 and 52.0 years, respectively. The median serum levels of HSPA1A were elevated in women with breast cancer (1037 pg/ml) compared with controls (300 pg/ml) (*p* < 0.001). Elevated HSPA1A levels were associated with advanced histological tumor grade (*p* < 0.001) and with the cell proliferation index (KI67) (*p* = 0.0418). The HSPA1A concentration was similar in women with different histological subtypes, nuclear grade, hormone receptor expression, HER2 status and the presence or absence of angiolymphatic invasion. Elevated serum HSPA1A in Brazilian women with advanced histological grade and proliferation index breast cancer supports the potential value of additional investigation on larger and more varied populations to verify the value of HSPA1A detection as a component of breast cancer diagnosis and progression.

## Introduction

The number of women diagnosed with breast cancer is increasing worldwide, and this malignancy is currently the second leading cause of death in women after cardiovascular disease. The World Health Organization (WHO) estimated an incidence of 9,227,484 new cancer cases in women and a mortality of 4,429,323 in 2020. Among these were 2,261,419 new cases of malignant breast cancer with a mortality of 684,996 women^1,2^. Malignant breast neoplasms, which are also one of the most common cancers among women in Brazil, second only to non-melanoma skin cancer, correspond to approximately 28% of new cancer cases each year. The National Cancer Institute in Brazil (INCA) estimated 316,280 new cancer cases in Brazilian women in 2020, with a mortality of 107,235 cases; of these, breast cancer was responsible for 66,280 new cases and 17,572 deaths^3^. The possibility of a cure for women with this malignancy is increased when the cancer is diagnosed at an earlier stage^2^. Therefore, better identification of variables associated with the initiation and evolution of this malignancy is needed for improvements in prevention, diagnosis and treatment.

Heat shock proteins are a family of proteins that are essential for the maintenance of cell homeostasis. Under physiological conditions, these proteins contribute to protein assembly, intracellular transport and the repair or degradation of misfolded proteins^4^. Heat shock proteins also regulate cellular metabolism, mitosis and apoptosis^5^. Under non-physiological adverse conditions, the intracellular level of the inducible 70 kDa heat shock protein (HSPA1A) becomes substantially elevated to preserve polypeptide structure and proper folding, inhibit programmed cell death and eliminate terminally damaged proteins. HSPA1A is also released from stressed cells and functions extracellularly to activate proinflammatory immunity^6^. Malignant transformation is one process that has been associated with elevated heat shock protein expression. Tumor cells typically express higher heat shock protein levels as a consequence of the hostile tumor cell environment due to deregulation of oncogenes and tumor suppressor genes as well as increased nutrient deprivation, hypoxia and acidosis. The increased heat shock protein expression in tumorigenesis not only allows cells to tolerate cumulative mutations and the expression of altered proteins, which would otherwise be lethal, but this increased expression also promotes cell survival through the inhibition of apoptosis^7–10^.

In view of the increasing incidence of malignant breast cancer and its high morbidity and mortality, new methods of diagnosis and treatment are currently being evaluated. It has been suggested that in women with breast cancer, serum levels of HSPA1A may be valuable as a diagnostic and predictive marker^11^. Elevated extracellular levels of HSPA1A have previously been identified in a number of malignancies^12–17^. An investigation of women with breast cancer concluded that elevated expression of HSPA1A was correlated with decreased disease-free survival^18^.

Due to a need to further explore the potential utility of HSPA1A measurement in breast cancer, coupled with the paucity of studies correlating its expression with breast cancer in Brazilian women, the objective of our study was to determine whether circulating HSPA1A levels could differentiate between women with malignant breast lesions and women without breast cancer. The Brazilian population is unique in its admixture of racial and ethnic groups from different continents over the centuries^19^. This mixing may have conferred unanticipated variations in HSPA1A expression and/or its transport to the extracellular milieu. While this study was not specifically designed to analyze variations in HSPA1A expression according to histological subtype, hormone receptor and HER2 expression, histological grade, nuclear grade, stage, cell proliferation index and the presence of lymphatic or vessel invasion, these possible associations were assessed on an exploratory basis.

## Methods

This exploratory, cross-sectional, analytical case–control study was approved by the Ethics Committee of the Cancer Institute of the State of São Paulo (project number 1035/2016) and by the Ethics Committee for Analysis of Research Projects at the Hospital das Clínicas of the USP Medical School (CAPPesq). Potential subjects were informed about the study at the time of a scheduled appointment, and if they agreed to participate and satisfied the inclusion and exclusion criteria, they provided written informed consent. All methods were performed in accordance with the relevant guidelines and regulations and were consistent with the Declaration of Helsinki.

Patient selection occurred between September 2017 and December 2018. During this period, all patients diagnosed with breast cancer who were seen at the First Consultation Clinic of the Cancer Institute of the State of São Paulo and who satisfied the inclusion criteria were invited to participate. Patients initially referred to the General Didactic Outpatient Clinic at the Mastology section at the Hospital das Clínicas of the USP Medical School because of abnormalities observed on a mammogram performed elsewhere, and who subsequently underwent a second mammogram with negative results at our center, and who met the inclusion criteria, were recruited for the control group.

The inclusion criteria of the breast cancer group were a histological diagnosis of breast cancer, no previous treatment for breast cancer, age between 25 and 75 years, the absence of signs or symptoms of other neoplasias and no previous history of other neoplasms. The exclusion criteria of the breast cancer group were the presence of noncarcinoma breast neoplasia, such as sarcoma or phyllodes tumor, and the presence of other comorbidities, such as nephropathies, liver disease, heart disease, hematopathologies, immunological diseases or other neoplasms. The inclusion criteria for the control group were women between 25 and 75 years of age, the absence of current signs or symptoms of other neoplasms and no previous history of neoplasms. The exclusion criteria for the control group were the presence of any neoplasia and the presence of other comorbidities, such as kidney disease, liver disease, heart disease, hematopathologies, immunological diseases or other neoplasms. Race was self-identified by each subject.

During the initial consultation, 10 ml of blood was collected in nonheparinized tubes and transported to the Structural and Molecular Research Laboratory in Gynecology at the Faculty of Medicine of the University of São Paulo within 30 min of collection. After clot formation, the serum fraction was collected by centrifugation and stored in aliquots at – 80 °C. Thawed serum was diluted 1:200 in phosphate-buffered saline-Tween 20 and tested for the concentration of HSPA1A using a commercial ELISA kit validated for human sera and specific for HSPA1A (R&D Systems, Minneapolis, MN). Each sample was tested in duplicate, and the average values were obtained. Values were converted to pg/ml by reference to a standard curve that was generated for each assay. The lower limit of sensitivity was 156 pg/ml. The demographic and clinical data of the patients participating in the study were obtained through consultation of electronic medical records.

Based on histopathological characteristics according to the WHO criteria^20^, breast cancer was classified as ductal carcinoma in situ, invasive carcinoma of no special type (invasive ductal carcinoma), invasive lobular carcinoma, and invasive mucinous carcinoma. The tumors were also classified into subtypes according to standard immunohistochemistry (IHC) findings. IHC was used to determine the expression of estrogen and progesterone receptors, HER2 expression and the level of Ki67^21,22^. Ki67 is a marker of cell proliferation and is expressed exclusively during active phases of the cell cycle. Therefore, higher Ki67 values indicate an elevated rate of cell proliferation. Additional characteristics were used to classify the tumors based on histological grade and nuclear grade according to the 8th edition of the TNM classification system^23^.

### Statistical analysis

In all patients the HSPA1A levels are described using the median value and interquartile range. Values between categories were compared using the Mann–Whitney test for variables with 2 categories or the Kruskal-Walli tests for variables with more than 2 categories. The Spearman rank correlation test was used to evaluate associations between the HSPA1A level and clinical and demographic characteristics. The generalized linear model (MLG) was used for the variables that presented descriptive levels below 0.2 in the unadjusted analyses (*p* < 0.2) and that had biological plausibility to influence the marker^24,25^. The present study was designated as exploratory due to the limited number of participants and, thus, was underpowered to assess differences in HSPA1A among subtypes of breast cancer lesions. The analyses were performed using IBM-SPSS for Windows version 22.0 software and tabulated using Microsoft-Excel 2010 software, and all tests were performed with a 5% significance level.

## Results

This study included 141 women, 14 of whom were excluded (6 from the control group and 8 from the breast cancer group) due to hemolysis of the serum samples. Therefore, 59 serum samples from controls and 68 serum samples from cancer patients were analyzed. Three of the breast cancer patients had ductal carcinoma in situ, while the remainder had invasive breast cancer, 51 at an early stage (1A and 2A) while 13 had locally advanced disease (> stage 2A). None had metastatic disease, and no subject was positive for BRCA mutations.

The sociodemographic parameters of the breast cancer patients and controls are shown in Table 1. No statistically significant differences were observed between groups in terms of age or body mass index. However, the racial distribution between the two groups was different. The control group had a higher proportion of White women (*p* = 0.0004), no Black women and a marginally lower proportion of women of mixed race (*p* = 0.0538) than the cancer group. In addition, the educational level was lower in the cancer group than in the control group (*p* < 0.0029).

**Table 1:** Sociodemographic factors of the study population.

Variable	Controls	Breast cancer	*p* value
	N = 59	N = 68	
Mean age in years (SD)	52.0 (13.1)	54.9 (9.3)	0.2967
Mean body mass index (kg/m^2^)	27.1 (5.6)	29.1 (6.2)	0.0765
**Race**			
White	50 (84.7%)	34 (54.8%)	0.0004
Black	0	9 (14.5%)	0.0029
Mixed	9 (15.3%)	19 (30.6%)	0.0538
**Education**			
Incomplete elementary grade	11 (19.0%)	45 (55.2%)	0.0003
Complete elementary grade	22 (37.9%)	8 (13.8%)	0.0029
Complete high school	13 (22.4%)	13 (22.4%)	1.000
Complete college	12 (20.7%)	5 (8.6%)	0.0678

**Figure 1:**
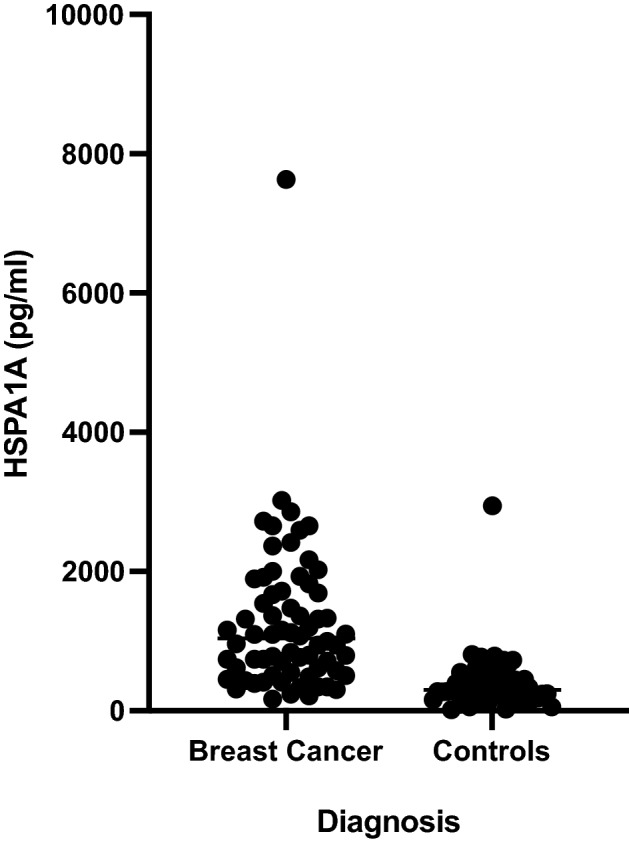
HSPA1A in serum samples from women with breast cancer and controls. Serum samples from 68 women with breast cancer and 59 controls were tested for HSPA1A by ELISA. The median (interquartile range) was 1037 (5601,713) pg/ml in breast cancer patients and 300 (192,521) in controls (*p* < 0.0001, Mann–Whitney test).

The HSPA1A level in the sera of individual breast cancer patients and controls is shown in Fig. 1. The median (interquartile range) value of HSPA1A was 1037 (5601,713) pg/ml in breast cancer patients and 300 (192,521) pg/ml in controls (*p* < 0.0001, Mann–Whitney test).

The HSPA1A levels in breast cancer patients and controls adjusted for race and age are shown in Table 2. In both premenopausal (26–49 years of age) and postmenopausal (≥ 50 years of age) women, HSPA1A levels were higher in White breast cancer patients than in controls (*p* ≤ 0.0064). In women of mixed race, HSPA1A concentrations were also higher in cancer patients than in controls for both age groups, but this difference did not reach statistical significance (*p* ≤ 0.0571). Within the cancer group, women ≥ 50 and of mixed race had the highest HSPA1A levels (*p* = 0.0183 vs. White women).

**Table 2:** HSPA1A in breast cancer patients and controls by race and age.

Diagnosis	Race	Age	No. Women	Median HSPA1A in pg/ml (Interquartile range)
Cancer	White	26–49	12	839 (362,1278)^a^
		≥ 50	25	892 (525,1326)^b,c^
	Black	26–49	2	754 (740,768)
		≥ 50	7	944 (396,2367)
	Mixed	26–49	6	1262 (1021,2014)^d^
		≥ 50	16	1825 (740,2655)^e^
Controls	White	26–49	21	275 (198,275)
		≥ 50	29	406 (212,546)
	Mixed	26–49	6	232 (151,610)
		≥ 50	3	559 (450,1092)

^a^*p* = 0.0064 vs. controls White 26–49; ^b^*p* < 0.001 vs. controls ≥ 50; ^c^*p* = 0.0183 versus cancer mixed ≥ 50; ^d^*p* = 0.0549 versus controls mixed 26–49; ^e^*p* = 0.0571 versus controls mixed ≥ 50.

Table 3 illustrates the serum HSPA1A levels in the cancer patients according to clinical stage and imaging findings. The 13 women with locally advanced breast cancer, > stage 2A disease, had the highest median HSPA1A level. However, this was not statistically different from the 55 women with in situ or early stage malignancy (stage 1A or 2A). In terms of mammographic findings, the 11 women with microcalcifications had an elevated median HSPA1A level (1201 pg/ml), as compared to median levels in women with asymmetrical lesions (614 pg/ml) (*p* < 0.0474). No differences were observed in HSPA1A levels between the 55 women with a single lesion, the 12 women with a multifocal lesion or the one subject whose lesion was multicentric.

**Table 3:** HSPA1A in women with breast cancer according to clinical stage and imaging findings.

Variable	No. women	Median HSPA1A (Interquartile range)
**Staging**		
In situ	3	1077 (437,1201)
Early stage (1A + 2A)	52	997 (507,1825)
Locally advanced (> 2A)	13	1159 (694,2189)
**Mammographic findings**		
Normal	2	1242 (311,2172)
Nodules	40	1097 (543,1792)
Microcalcifications	11	1201 (944,1930)^a^
Asymmetry	11	614 (507,1100)
Architectual distortion	4	1073 (661,1779)
**Type of lesion**		
Single	55	997 (614,1545)
Multifocal	12	1052 (478,2289)
Multicentric	1	1669

^a^*p* = 0.0474 versus asymmetrical lesions (Mann–Whitney test).

Serum values of HSPA1A in relation to anatomopathological and immunohistochemical findings are presented in Table 4. The HSPA1A level increased proportionally with the histological grade of the tumor (*p* < 0.001). In contrast, the median HSPA1A level was not significantly different in women diagnosed with various histological subtypes. The HSPA1A level was increased in proportion to the cell proliferation index (Ki-67), yielding a median value of 1340 pg/ml when the index was > 30 as opposed to values of 943 pg/ml and 579 pg/ml when the index was 15–30 and < 15, respectively *p* = 0.0418). The concentration of serum HSPA1A was unrelated to the presence or absence of angiolymphatic invasion, estrogen or progesterone receptor expression or HER2 status.

**Table 4:** HSPA1A in women with breast cancer according to anatomopathological and immunohistochemical findings.

Variable	No. women	Median HSPA1A (Interquartile range)
**Histological subtype**		
Ductal carcinoma in situ	3	740 (337,1330)
Mucinous carcinoma	4	526 (364,961)
Invasive ductal carcinoma	55	1097 (579,1693)
Invasive lobular carcinoma	6	1768 (542,2706)
**Histological grade**		
1	11	716 (337,944)
2	38	776 (478,1199)
3	19	1719 (1201,2172)^a^
**Nuclear grade**		
2	31	997 (579,1719)
3	37	1097 (531,1795)
**Angiolymphatic invasion**		
Absent	49	997 (600,1694)
Present	15	1024 (452,3632)
**Estrogen receptor**		
Negative	12	1367 (597,2028)
Positive	56	962 (531,1681)
**Progesterone receptor**		
Negative	16	1367 (597,1821)
Positive	52	892 (507,3021)
**HER2**		
Positive	12	1262 (518,1527)
Negative	52	953 (507,1707)
**Ki-67 (%)**		
< 15	13	579 (450,1099)
15–30	12	943 (751,1278)
> 30	40	1340 (712,1983)^b^

^a^*p* ≤ 0.0001 (Kruskal–Wallis test); ^b^*p* = 0.0418 (Kruskal–Wallis test).

In women with invasive breast cancer, no association was found between tumor diameter as assessed by mammography and the circulating HSPA1A level (Table 5).

**Table 5:** HSPA1A in women with invasive breast cancer according to tumor size.

Tumor size (cm)	No. women	Median HSPA1A (Interquartile range)
< 2	20	953 (432,1327)
2–5	34	1112 (619,1726)
> 5	7	1077 (579,1363)

Tumor size was determined by mammography.

## Discussion

In our initial exploratory study of Brazilian women on their first visit to a breast cancer clinic, serum levels of HSPA1A were greatly elevated in those with breast cancer as compared to those with no breast malignancy. In addition, among breast cancer patients significantly elevated levels were observed in those with the highest histological grade lesion and the highest cell proliferation index. Perhaps due to the limited sample size, no other statistically significant differences were observed in HSPA1A in relation to breast lesion characteristics or hormone receptor status. Our findings in Brazilian women, despite representing a limited number of subjects and the absence of confirmatory messenger RNA detection, parallel more detailed reports from other populations on the association between HSPA1A gene expression and breast cancer^11,12^ and the absence of an association between HSP1A expression and the size of the mammary tumor^18^. In addition, an investigation of more than 3000 breast cancer specimens revealed that the messenger RNA coding for HSPA1A was highly over-expressed in these malignant tissues^26^. Elevated HSPA1A gene expression in tumor tissues is apparently not limited to breast cancer. A study in individuals with colon cancer demonstrated that elevated HSPA1A expression was correlated with poor prognosis^27^.

It is known that highly undifferentiated breast tumors (histological grade 3) are the most aggressive, have greater lymph node involvement and have a greater capacity for cell replication (high KI67 index). The finding of the highest HSPA1A levels in breast cancer patients with these characteristics suggests that HSPA1A production and release into the circulation may be an indication of these factors. Our observations that the serum level of HSPA1A was lower in women with carcinoma in situ than in those with locally advanced invasive carcinomas and lower in well-differentiated slowly proliferating tumors than in more rapidly growing undifferentiated tumors are complementary to a prior analysis of intracellular HSPA1A levels in samples of breast cancer tissue^28^. These authors noted that increased expression of HSPA1A was related to decreased cell differentiation, that is, to tumors with a higher histological grade. Similarly, Vargas-Roig et al.^29^ found a correlation between increased HSPA1A expression in breast carcinoma tissue biopsies and an increased mitotic index. The concordance of our extracellular findings with these intracellular analyses suggests that determination of HSPA1A levels in the extracellular milieu may be a less invasive method to evaluate these tumor-related variables. However, in a systematic review, Dimas et al.^18^ found no relationship between the histological grade of breast tumors and the intracellular expression of HSPA1A. Additional studies are needed to determine whether population and/or racial variations influence these associations. Our study did not contain a follow-up component, and thus, we were unable to evaluate possible associations between the HSPA1A level and the development of recurrent or distant disease or survival.

An evaluation of hormone receptor and HER2 status is essential to optimize breast cancer treatment for individual patients. Surprisingly, only a few studies have evaluated the association between HSPA1A status and hormone receptor status in breast cancer. Takahashi et al.^30^, after evaluating surgical samples of breast cancer tissue, reported higher HSPA1A positivity in estrogen receptor-positive tumors. Regarding progesterone receptors, Lazaris et al.^27^ found a correlation between increased HSPA1A and the presence of the progesterone receptor. Dimas et al.^18^ similarly identified a correlation between an increase in HSPA1A and the presence of hormone receptors but found no correlation between HSPA1A and HER2. In the present study, no differences were identified between serum levels of HSPA1A in women with breast cancer and the expression of these hormone receptors. It is possible that the differential release of HSPA1A from mammary tumors that are positive or negative for these receptors is too small to be detected in the systemic circulation. Differences in the populations studied may also contribute to the observed outcome variations.

Elevated extracellular expression of HSPA1A in women with breast cancer, in addition to being a marker of malignancy, suggests that this heat shock protein may participate in the initiation and/or persistence of carcinogenesis. While intracellular HSPA1A levels in tumor tissues were not evaluated in our study, it is well known that extracellular HSPA1A levels reflect elevated intracellular concentrations^6^. Intracellular HSPA1A can inhibit cell apoptosis and interrupt the senescence process, which are two mechanisms that are central to the prevention of unrestrained cell division and whose inhibition can lead to tumor formation ^5,9,31^.

## Conclusions

In this exploratory investigation serum levels of HSPA1A were increased in Brazilian women with breast cancer and there was a relationship between the HSPA1A concentration and advanced tumor characteristics. The findings were consistent with studies on other populations and strongly suggest that further studies of serum HSPA1A in breast cancer are warranted and have the potential to contribute to improved clinical diagnoses, prognosis and treatment of this prevalent malignancy of women.

## Data Availability

All relevant data is included in the manuscript. Correspondence and requests for materials or additional information should be addressed to Dr. Gabriela Boufelli de Freitas at gboufellifreitas@gmail.com.
